# Nonlinear Associations between Medical Expenditure, Perceived Medical Attitude, and Sociodemographics, and Older Adults’ Self-Rated Health in China: Applying the Extreme Gradient Boosting Model

**DOI:** 10.3390/healthcare10010039

**Published:** 2021-12-26

**Authors:** Yuqing Liang, Wanwan Zheng, Woon-Seek Lee

**Affiliations:** Graduate School of Management of Technology, Pukyong National University, 365 Sinseon-ro, Nam-gu, Busan 48547, Korea; liangyuqing@pukyong.ac.kr (Y.L.); zhengwanwan@pukyong.ac.kr (W.Z.)

**Keywords:** medical expenditure, perceived medical attitude, extreme gradient boosting

## Abstract

Background: although China’s total health expenditure has been dramatically increased so that the country can cope with its aging population, inequalities among individuals in terms of their medical expenditures (relative to their income level) have exacerbated health problems among older adults. This study aims to examine the nonlinear associations between each of medical expenditure, perceived medical attitude, and sociodemographics, and older adults’ self-rated health (SRH); it does so by using data from the 2018 China Family Panel Studies survey. Method: we used the extreme gradient boosting model to explore the nonlinear association between various factors and older adults’ SRH outcomes. We then conducted partial dependence plots to examine the threshold effects of each factor on older adults’ SRH. Results: older adults’ medical expenditure exceeded their overall income. Body mass index (BMI) and personal health expenditure play an essential role in predicting older adults’ SRH outcomes. We found older adult age, physical exercise status, and residential location to be robust predictors of SRH outcomes in older adults. Partial dependence plots of the results visualized the nonlinear association between variables and the threshold effects of factors on older adults’ SRH outcomes. Conclusions: findings from this study underscore the importance of medical expenditure, perceived medical attitudes, and BMI as important predictors of health benefits in older adults. The potential threshold effects of medical expenditure on older adults’ SRH outcomes provide a better understanding of the formation of appropriate medical policy interventions by balancing the government and personal medical expenditure to promote health benefits among older adults.

## 1. Introduction

Alongside China’s social and economic development, its population is growing at a rate faster than that seen in almost any other country. According to the United Nations World Report on Population Aging 2019 [1], the percentage of China’s population aged over 60 years is projected to reach 28% by 2040, as lifespans are expected to increase and fertility rates to decline. This dramatic demographic shift poses new challenges in (and opportunities for) improving public health. In particular, during the COVID-19 pandemic, public healthcare and related interventions have been required, and under such circumstances, knowledge of the social determinants of healthy aging has been crucial to establishing evidence-based policies and interventions by which to improve older adults’ health benefits.

Although China’s total health expenditure has dramatically increased in recent decades due to its aging population, the individuals within that demographic still face financial difficulties on account of health problems. One underlying reason for this is that their wealth growth cannot cover medical expenditures. According to research findings [2], the health expenditure growth rate during the 2010–2013 period was 13.2%—a figure 1.62 times higher than the rate gross domestic product growth during the same period. Although China’s high medical insurance coverage levels have played an essential role in mitigating the related financial burden, many residents still have limited access to healthcare [3]. Those without medical insurance and access to universal healthcare still need to make out-of-pocket payments, many of which can be high. Such an imbalance among rapid medical expenditure growth, limited healthcare accessibility, and out-of-pocket payments poses a new challenge for the government as it looks to maintain healthcare balance and promote the people’s health and wellbeing, especially among vulnerable groups such as children and elderly people [4]. 

The literature indicates that medical expenditure plays a core role in influencing health benefits. For instance, research has found a significant association between medical expenditure and chronic diseases in the Korean population [5]. Another study, conducted in China, found that healthcare expenditure is associated with obesity [6]. In terms of specific health outcomes, one study indicates that higher medical expenditure and lower quality of life can best predict comorbidity among patients with acute coronary syndrome [7]. Although these findings provide consistent evidence of the linear or nonlinear association between medical expenditure and health outcomes, findings on the degree to which medical expenditure contributes to people’s health benefits are not conclusive. A major limitation of most studies on such associations is that they fail to address the potential threshold effect of medical expenditure on people’s health benefits. It is important for the government to allocate appropriate medical expenditure and healthcare resources rationally, especially for vulnerable residents who do not have medical insurance and live in underserved areas—areas where residents often face significant care barriers owing to a lack of health services access [8,9].

Individuals’ subjective assessments of medical services have been considered another essential measure of healthcare quality, a core factor affecting people’s health outcomes. Studies indicate a significant association between medical services satisfaction and various health outcomes (e.g., depression, psychological wellbeing, mental health, and self-rated health [SRH]) [10]. Specifically, one study found that people who were more satisfied with the quality of healthcare services are less likely to perceive of depression symptoms [11]. In addition, other studies found a mixed association between trust in individuals and healthcare systems. One study, for example, indicates a significant association between trust in physicians and health symptoms [12]; another found a linear and curvilinear association between trust in doctors and anxiety [13]. This trust-based association is developed through physicians’ execution of “medical expertise” [14], something considered a major source of anxiety that warrants further investigation [15]. Elsewhere, the use of medical services has been associated with occurrences of panic disorder and generalized anxiety disorder [16]. Social security has also affected people’s psychological wellbeing, as China has experienced in recent years a dramatic increase in the number of medical dispute cases [17]. Another limitation inherent in most published studies is that while they indicate significant correlations among health benefits, they do not identify those subjective factors that best predict individual-level health benefits.

Additionally, a variety of studies show that individuals’ social characteristics and health risk factors correlate with health outcomes. For instance, research findings consistently reveal that female respondents are more likely to report a lower level of SRH compared to male counterparts [18]. Such effects are more pronounced among older women who experience socioeconomic disadvantages [19]. In addition, studies have found that older respondents are more likely to report a lower SRH than younger respondents [20], and respondents with a higher educational attainment level or who are married were more likely to perceive better SRH [21]. Additionally, respondents who are current employees or early retirees are less likely to report poor SRH than those who do not satisfy these criteria [22], and higher-income respondents are more likely to perceive better SRH [23]. Respondents with urban *hukou* are more likely to achieve good SRH outcomes than those without urban *hukou* [24] while other studies found opposite *hukou*–SRH associations [25]. Respondents with chronic disease tend to have a higher probability of poor SRH. Poor SRH tends to emerge among respondents who are current smokers or drinkers, while those with sufficient physical activity in the previous week tend to report good SRH [26,27,28]. Finally, obese respondents are more likely to report lower health benefits than those with a normal weight [29]. 

Despite the growing interest in examining the association between each of medical expenditure, perceived medical attitude, and sociodemographic characteristics, and SRH among older adults, few studies identify those factors that most impact older adults’ SRH, and there is little information about the threshold effects of various factors on older adults’ SRH. Accordingly, this study examines the importance of various factors on older adults’ SRH and further explores the potential threshold effect of those factors on older adults’ SRH; it does so by using a machine-learning approach. This study contributes to a comprehensive understanding of the importance and threshold effects of various factors that affect the health of older adults in China. 

## 2. Materials and Methods

### 2.1. Sample Description

The data used in this study were derived from the China Family Panel Studies (CFPS) survey, a national and longitudinal survey covering 25 provinces in China. These data were collected through the Institute of Social Science Survey at Peking University, following a stratified multistage sampling strategy. The sample therein consists of 95% of the Chinese population, and so it can be considered nationally representative.

The baseline survey of the CFPS was first launched in 2010, and four full-sample follow-up surveys occurred during the 2012, 2014, 2016, and 2018 waves. The CFPS paid special attention to investigating the economic and noneconomic wellbeing of Chinese individuals and covered a wide range of research areas, including social activities and attitudes, sociodemographic characteristics, and physical and mental health.

In this study, we use the 2018 CFPS database because it provides a sufficient set of questions by which to measure individual-level sociodemographic characteristics and health outcomes. Five major questionnaires were designed as part of the CFPS: a community questionnaire, a family roster questionnaire, a family questionnaire, a child questionnaire, and an adult questionnaire. In accordance with our research objective, we used data only from the adult and family questionnaires. 

### 2.2. Data Acquisition

First, we matched two databases and removed duplicate records, thus capturing a sample of 32,669 records. Second, we excluded those records with responses of “I don’t know,” “not applicable,” or “I refuse to answer,” as well as those with missing values. Third, we excluded the records of individuals aged under 60 years. Ultimately, we derived a sample comprising 4864 records to explore various predictors of older adults’ SRH.

### 2.3. Variables Definition

#### 2.3.1. Outcome

The outcome of this study is SRH, which has been widely used as a comprehensive assessment of individual-level health status stability [30,31]. It is measured by posing a question: “How would you rate your health status?” Responses to this question are captured using a five-point Likert scale, with reported values of 1 (“excellent”), 2 (“very good”), 3 (“good”), 4 (“fair”), and 5 (“poor”). We converted these values into dummy variable values, with an SRH of 1–4 considered good and an SRH of 5 considered poor. 

#### 2.3.2. Medical Expenditure and Perceived Medical Attitudes

In the current study, one of the core explanatory predictors of SRH is the individual-level cost of healthcare. We measured individuals’ total medical expenditure by posing the following question: “In the past 12 months, how much money has been spent on the total direct medical expenditure (excluding what was reimbursed or reimbursable but including what was paid by or borrowed from relatives)?” We coded this item as a continuous variable. Another essential predictor of SRH is perceived medical attitude, which we measured by posing the following question: “How would you rate the severity of the medical service problem in China?” Response values ranged from 0 (“no problem”) to 10 (“extremely serious problems”). Attitudes pertaining to social security problems were measured by posing the question: “How would you rate the severity of the social security problem in China?” Response values ranged from 0 (“no problem”) to 10 (“extremely serious problems”). Trust in doctors was measured with the question “How much do you trust doctors?” Response values ranged from 0 (“distrustful”) to 10 (“very trusting”). Finally, attitude regarding medical expertise was measured with the question “What do you think of the medical expertise level?” Response values ranged from 1 (“very bad”) to 5 (“very good”). 

#### 2.3.3. Related Health Risk Factors and Sociodemographic Characteristics

Studies indicate that individuals’ physical health status, physical activity, and daily behaviors significantly correlate with their SRH. Accordingly, we explored five related health risk factors that would play an important role in affecting SRH—namely, BMI, chronic disease, frequent physical activity, and current smoking or drinking. BMI was measured by asking respondents about their current height and weight; responses were continuous variables. Chronic disease status was measured with the question “During the past six months, have you had any doctor-diagnosed chronic disease?” We coded the answers as 1 (“yes”) or 0 (“no”). Frequency of physical activity was measured with the question “How often did you participate in physical exercise in the past week?”. Responses were continuous variables. Smoking status was measured with the following question: “Did you smoke cigarettes in the past month?” We coded the answer as 1 (“yes”) or 0 (“no”). We measured drinking status using the following question: “Did you drink alcohol at least three times a week in the past month?” This item was coded as 1 for drinkers and 0 otherwise. Finally, we adjusted for sociodemographic characteristics such as age, gender, marital status, educational attainment level, household income level, family size, employment status, and *hukou* status; we also considered whether respondents were retired, living in an urban area, or had medical insurance. Note that *hukou* status refers to a salient social identity in China where respondents who live in urban areas are categorized as urban residents, while respondents living in rural areas are categorized as rural residents. Economic resources, education, employment, and social welfare benefits are privileged among urban residents [32].

### 2.4. The Extreme Gradient Boosting Model

In this study, we apply the extreme gradient boosting (XGBoost) model to explore the possible nonlinear association between various predictors and SRH in older adults. XGBoost was proposed by Chen and Guestrin [33] and is an improved and developed model based on the gradient boosted decision trees (GBDT). (The GBDT model had, in turn, been developed based on the tree learning method and is used to predict data.) Recently, studies have used the GBDT model to examine the nonlinear association between the built environment and travel behaviors [34,35]. The main difference between the GBDT and XGBoost models is that the former uses only first-order Taylor expansion, whereas the latter performs second-order Taylor expansion on the loss function. More importantly, XGBoost also adds the LASSO (L1) regularization term and the ridge (L2) regularization term to penalize more complex models, to control model complexity and prevent model overfitting. XGBoost also helps improve the generalization ability of the model.

The XGBoost model was developed into a package and launched on several platforms, such as Python and R. In this study, we ran the package in Python 3.6. We present only the key equation of XGBoost; further details can be found in [33].

The loss function is defined as:

(1) $$Obj=\overset{n}{\underset{i=1}{\sum }}l\left(y_{i},{\hat{y}}_{i}\right)+\overset{K}{\underset{K=1}{\sum }}\Omega \left(f_{k}\right)$$

where l refers to the convex loss function, which measures the difference between the prediction and target objectives. Meanwhile, Ω denotes the term that penalizes the complexity of the model. Here,

(2) $$\Omega \left(f\right)=\gamma T+\frac{1}{2}\lambda ∥w_{i}{∥}^{2}$$

where T is the number of leaves in the tree, $w_{i}$ is the term that represents the score on the *i*th leaf, and γ and λ denote regularization parameters.

The objective function can be expanded by greedily adding $f_{t}$ as:

(3) $$Obj^{\left(t\right)}=\overset{n}{\underset{i=1}{\sum }}l\left(y_{i},{\hat{y}}_{i}^{\left(t-1\right)}+f_{t}\left(x_{i}\right))+\Omega f_{t}\right)+constant$$

According to Taylor expansion, the equation can be rewritten as:

(4) $$Obj^{\left(t\right)}⋍\overset{n}{\underset{i=1}{\sum }}\left[l\left(y_{i},{\hat{y}}_{i}^{\left(t-1\right)}+g_{i}f_{t}\left(x_{i}\right)+\frac{1}{2}h_{i}f_{t}^{2}\left(x_{i}\right)\right)+\Omega \left(f_{t}\right)\right]$$

where $g_{i}$ and $h_{i}$ denote the first and second derivations on the loss function, respectively:

(5) $$g_{i}=\partial_{{\hat{y}}_{i}{}^{\left(t-1\right)}}l\left(y_{i},{\hat{y}}_{i}{}^{\left(t-1\right)}\right)$$

(6) $$h_{i}=\partial_{{\hat{y}}_{i}{}^{\left(t-1\right)}}^{2}l\left(y_{i},{\hat{y}}_{i}{}^{\left(t-1\right)}\right)$$

We removed the constant term and optimized the objective at step *t* as:

(7) $$Obj^{\left(t\right)}=\overset{n}{\underset{i=1}{\sum }}\left[g_{i}f_{t}\left(x_{i}\right)+\frac{1}{2}h_{i}f_{t}^{2}\left(x_{i}\right)\right]+\Omega \left(f_{t}\right)$$

We further define $I_{j}=\left\{i|q\left(x_{i}\right)=j\right\}$ as the instant set of leaf j. We can obtain the optimal value of $\omega_{i}^{*}$ by solving the above equations. Here,

(8) $$\omega_{i}^{*}=-\frac{\sum_{i\in I_{j}}\partial_{{\hat{y}}_{i}{}^{\left(t-1\right)}}l\left(y_{i},{\hat{y}}_{i}{}^{\left(t-1\right)}\right)}{\sum_{i\in I_{j}}\partial_{{\hat{y}}_{i}{}^{\left(t-1\right)}}^{2}l\left(y_{i},{\hat{y}}_{i}{}^{\left(t-1\right)}\right)+\lambda }$$

(9) $${\tilde{Obj}}^{\left(t\right)}\left(q\right)=-\frac{1}{2}\overset{n}{\underset{j=1}{\sum }}\frac{{\left(\sum_{i\in I_{j}}\partial_{{\hat{y}}_{i}{}^{\left(t-1\right)}}l\left(y_{i},{\hat{y}}_{i}{}^{\left(t-1\right)}\right)\right)}^{2}}{\sum_{i\in I_{j}}\partial_{{\hat{y}}_{i}{}^{\left(t-1\right)}}^{2}l\left(y_{i},{\hat{y}}_{i}{}^{\left(t-1\right)}\right)+\lambda }+\gamma .$$

Note that equation x can be used to measure the quality of tree structure q. However, in a real-world situation, it is impossible to calculate all the possible tree structures q. Therefore, we propose a greedy algorithm that adds branches to the tree, which begins with a single tree. We split the trees into the left nodes ($I_{L}$) and right nodes ($I_{R}$). Finally, we let $I=I_{L}\cup^{\;}I_{R}$, where the loss function can be written as:

(10) $$Obj_{split}=\frac{1}{2}[\frac{{\left(\sum_{i\in I_{L}}\partial_{{\hat{y}}_{i}{}^{\left(t-1\right)}}l\left(y_{i},{\hat{y}}_{i}{}^{\left(t-1\right)}\right)\right)}^{2}}{\sum_{i\in I_{L}}\partial_{{\hat{y}}_{i}{}^{\left(t-1\right)}}^{2}l\left(y_{i},{\hat{y}}_{i}{}^{\left(t-1\right)}\right)+\lambda }+\frac{{\left(\sum_{i\in I_{R}}\partial_{{\hat{y}}_{i}{}^{\left(t-1\right)}}l\left(y_{i},{\hat{y}}_{i}{}^{\left(t-1\right)}\right)\right)}^{2}}{\sum_{i\in I_{R}}\partial_{{\hat{y}}_{i}{}^{\left(t-1\right)}}^{2}l\left(y_{i},{\hat{y}}_{i}{}^{\left(t-1\right)}\right)+\lambda }-\frac{{\left(\sum_{i\in I}\partial_{{\hat{y}}_{i}{}^{\left(t-1\right)}}l\left(y_{i},{\hat{y}}_{i}{}^{\left(t-1\right)}\right)\right)}^{2}}{\sum_{i\in I}\partial_{{\hat{y}}_{i}{}^{\left(t-1\right)}}^{2}l\left(y_{i},{\hat{y}}_{i}{}^{\left(t-1\right)}\right)+\lambda }]-\gamma$$

### 2.5. Model Evaluation

To ascertain whether XGBoost offers good performance and accuracy in predicting older adults’ SRH, we leveraged various models—including random forest, LightGBM, and logistic regression models—and compared the results thereof. Each model was evaluated in terms of precision, recall, F1 score, area under the curve (AUC), and R-square. Precision denotes the proportion of correctly predicted positive observations in all predicted observations, and recall refers to the proportion of correctly predicted positive observations for all observed positive observations. The F1 score represents the harmonic mean of the precision and sensitivity. Finally, accuracy denotes the accuracy of the model. The overall results indicate that the XGBoost had the best prediction performance, with a precision of 0.7909, recall of 0.8920, F1 score of 0.8385, accuracy of 0.7515, and AUC of 0.6386. In addition, the XGBoost model achieved a relatively good fit, with an R-square value of 0.92. 

The dataset was first split into training sets (80%) and testing sets (20%), followed by a grid search with five-fold cross-validation launched from a sklearn model selection (https://scikitlearn.org/stable/modules/generated/sklearn.model_selection.GridSearchCV.html (27 October 2021) to capture the best-performing hyperparameters. Furthermore, the training set was randomly distributed into five subsamples, four of which were used for training; the remaining subsample was used to validate each model.

For the search process, the model was first run with a default search value derived from the XGBoost 1.5.0-dev documentation. The search process was initiated by searching for the best value within a range of estimator numbers, which was set to 100–1000. The best score was obtained when the number of estimators was 200. This was followed by a search process wherein we set *min_child_weight* (range of 1–10) and *maximum* *depth* (range of 1–10); the model achieved the best score when *min_child_weight* and *max depth* were set to 8 and 2, respectively. The best model score occurred when the *gamma* parameter (range of 0.1–1.0) equaled 0.9. The *subsample* (range of 0.3–1.0) and *colsample_bytree* (range of 0.3–1.0) were the next search parameters, and we found that the model achieved the best score when these were set to 0.5 and 0.4, respectively. The parameters *reg_alpha* and *reg_lamba* were selected from the candidates, and both ranged from 0.05 to 4; the best score was achieved when *reg_alpha* was set to 0.05 and *reg_lamba* to 1. Finally, the optimal value of the learning rate was searched (range of 0.01–0.1), and we captured the best model score when the learning rate was set to 0.1.

## 3. Results

Table 1 shows the current study’s baseline factors. Note that 72.1% of the respondents (N = 4864) reported a good SRH, while 28.1% of them reported having a chronic disease. There were slightly more male respondents (57%) than female ones (43%); additionally, 87% of all respondents were married, 16.6% had *hukou*, 36.5% were urban residents, and 77.7% were employed. The average age of the respondents was 66 years. Only 11.3% of the respondents were retirees, and 65.2% had medical insurance. Interestingly, the average total income of the respondents was 3624 yuan per year, while the average total personal expenditure on medical expenses exceeded 4600 yuan per year. These numbers indicate that approximately 35% of respondents lacking medical insurance might run behind on their medical expenses. Regarding health risk characteristics, the average frequency of physical exercise for respondents was three times per week, 49.9% of respondents were current smokers, and 20.7% of respondents had consumed alcohol at least three times a week in the previous month. The average BMI of respondents was 22.8; this value is consistent with the normal weight for older adults in Asian countries [36]. In terms of the subjective assessment of medical services, over 70% of respondents trusted doctors, over one-half of the respondents were satisfied with the medical expertise at hospitals, and 50% of the respondents had a neutral attitude towards medical service and social security problems. 

### 3.1. Importance of Various Factors on Older Adult’s Self-Rated Health

Table 2 shows the importance of various factors in predicting older adults’ SRH, using various feature-ranking measures. In column 1, the factors are ranked in accordance with F-score feature importance. The F-score denotes the selection method that examines the association between each descriptive factor and the target outcome, derived by using the F-distribution. However, the literature shows that using the F-score as the only feature selection method is not sufficient, as it does not reveal mutual information among features [37]. Accordingly, we applied Shapley additive extensions (SHAP) to justify the robustness of the feature ranking (column 2). As noted in [38], each feature can be assigned an essential value by SHAP; this value speaks to the effect of including that feature in model prediction. The importance of each feature can be measured by computing the absolute Shapley values for each factor. 

The results in Table 2 suggest that BMI and personal health expenditure each plays an essential role in predicting older adults’ SRH. Subjective assessments of medical attitudes ranked moderately, in addition to the satisfaction with level of medical expertise; these findings denote the importance of the subjective assessment of medical attitudes in predicting older adults’ SRH. Age, physical exercise status, and residential location were robust factors predicting older adults’ SRH, while factors such as gender, family size, employment status, and chronic disease showed unstable performance in predicting older adults’ SRH when using various measures of feature ranking. Sociodemographic characteristics (e.g., marital status, retirement status, and residential location) have limited predictive power vis-à-vis older adults’ SRH. 

### 3.2. Association between Factors and Older Adults’ Self-Rated Health

To ascertain the potential threshold of factors that can impinge upon older adults’ SRH, we used using partial dependence plots (pdps) to examine correlations between various factors and older adults’ SRH. Studies indicate that pdps can visualize the marginal effects of factors on the outcome [39]. 

**Figure 1:**
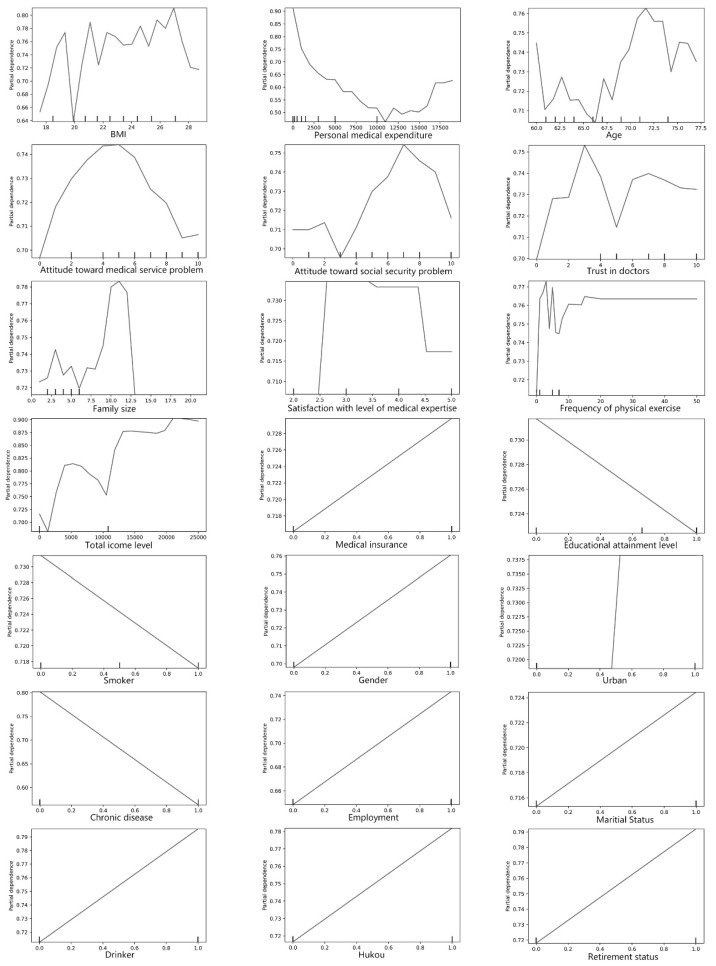
Nonlinear association between factors and older adults’ SRH (Note: Y-axes represent log-odds).

Figure 1 shows the effects of various factors on older adults’ SRH. In that figure, the X-axes represent each factor, while the Y-axis represents the logarithm of odds of SRH (i.e., the probability of reporting good SRH divided by the probability of reporting poor SRH). We found a nonlinear association between BMI and SRH in older adults: the SRH of older adults increased when BMI reached 20 and began to decline when BMI reached 27. This is plausible, as older adults often suffer from being either underweight or overweight, either of which can result in poor health status [40,41]. As expected, we found a negative association between medical expenditure and SRH. Specifically, the probability of reporting good SRH decreased sharply when medical expenditure was lower than 11,000 yuan; it then increased slightly thereafter. We observed an approximate V-shaped fluctuation between age and SRH, with a potential threshold of reporting good SRH at age 66. Nonlinearity and threshold effects existed in terms of the association between perceived medical attitudes and SRH. Overall, older adults’ SRH decreased with an increase in the perception of negative medical attitudes. Regarding health risk factors, we found that older adults were more likely to report the best health outcomes when family size was reportedly 10. The positive effect of the frequency of physical exercise on older adults’ SRH no longer increased when the frequency of physical exercise exceeded 18 times a week. Older adults who were current smokers and had a chronic disease were more likely to report poor SRH; however, we found a positive association between drinking status and SRH in older adults. Finally, in terms of sociodemographic characteristics, we found that older adults who were male, married, employed, retired, or urban residents, as well as those with local *hukou*, a high income level, and medical insurance, tended to report good SRH; those who had a higher educational attainment level were more likely to report poor SRH. 

## 4. Discussion

In implementing the Healthy China 2030 national strategy, the Chinese government has in recent decades gradually launched a series of healthcare reforms. However, the imbalance between out-of-pocket medical expenditure and income level poses a serious threat to health and wellbeing among China’s citizens; this is especially true for vulnerable populations with limited access to healthcare. Therefore, this study explored the nonlinear association between each of medical expenditure, perceived medical attitude, and sociodemographic factors and older adults’ SRH using the XGBoost model and pdps. This study provides new empirical evidence that policymakers and intervention designers can leverage to generate appropriate healthcare policies, with a special focus on balancing health expenditure and individuals’ medical expenditure and promoting older adults’ SRH. 

The results of our statistical analyses reveal that older adults’ medical health expenditure generally exceeded their income level; this suggests that the majority of older adults might suffer from a shortage of medical resources. This finding conflicts with the national record, which suggests that in 2018, 95% of the population was covered by health insurance [42]. One potential explanation might be that older adults who are unemployed are not fully covered by basic health insurance. Meanwhile, governments might consider adjusting the healthcare system to mitigate the financial burden of health problems, while paying special attention to those who do not have medical insurance and have limited access to healthcare. Regarding results pertaining to the importance of factors effecting SRH, we found that BMI and medical expenditure had the greatest predictive power in predicting older adults’ SRH, relative to the other factors studies. These results provide new insights into the core factors that contribute to predicting the health benefits of older adults. Additional findings indicate that the effects of subjective assessments of medical attitudes on older adults’ SRH should not be overlooked. 

Regarding the pdp results, we found a nonlinear relationship between medical expenditure and SRH. An 11,000-yuan personal medical expenditure might be a potential threshold at which older adults’ health benefits attenuate. This finding sheds light on the importance of refining medical expenditure as a medical expense, for in the current context it considerably impedes equitable healthcare access. The results also suggest that older adults with a BMI level lower than 18 or higher than 26 are more likely to report poor SRH; this finding is consistent with the finding that being underweight and having severe obesity is significantly associated with poor health status [41]. Furthermore, we found an overall negative association between perceived medical attitudes and older adults’ SRH. This finding is intuitive, as negative attitudes tend to associate with poor health status [43]. In addition, we found a positive association between the frequency of physical exercise and older adults’ SRH, but with a threshold of 18 times per week. This finding is partially consistent with those of other studies that suggest that older adults reporting 150 min per week of self-reported physical exercise tend also to report better psychological wellbeing, relative to those who do not [44,45]. Nevertheless, there does not appear to be a specific threshold for health benefits accruing to physical exercise that is recommended for older adults [46]. Consistent with the findings of other studies, we found that older adults who smoke and have a chronic disease are more likely to report poor SRH [47,48]. Older adults who are urban residents and have local *hukou* status are more likely to report good SRH [49,50]; one possible explanation for this urban–rural divergence might be that older adults who live in rural areas suffer from unequal access to resources relative to their urban counterparts, given that rural China generally has limited infrastructure accessibility [51]. Health insurance in rural and urban areas may differ in terms of the number and quality of healthcare facilities in each, and this asymmetry exacerbates health disparities [52].

Although this study offers substantial insights that can inform healthcare interventions and policies by which to promote health benefits among older adults, a few study limitations should be noted. First, given the cross-sectional nature of the data we used, we ignored the causal relationship between various factors and older adults’ SRH. Older adults with better SRH might be more likely to afford medical expenditure and thus have a better attitude toward medical services. Future longitudinal studies that leverage comprehensive medical data (in addition to CFPS data) should examine causal relationships. Second, future research should examine specific health indicators when such data are available, as SRH-oriented questions might invoke recall bias that would affect the results. Finally, although the XGBoost model provides a better understanding of the nonlinear association between various characteristics and health benefits, we were unable to explore significance between variables; this limitation stems from the nature of the XGBoost attributed to the black-box model, and it warrants further study. Deep learning techniques might be another suitable route to quantitative insights that predict individuals’ health benefits, once quantifiable spatial data are available. Future studies could also explore the deeper application of potential mechanisms in neural networks, such as CNNS [53], FCN-8s [54] and LSTM [55].

## 5. Conclusions

This study explored nonlinear associations between each of personal medical expenditure, perceived medical attitude, and sociodemographics, and self-rated health (SRH) outcomes, with a special focus on older adults, a somewhat vulnerable population. We used a specific machine-learning approach (i.e., the extreme gradient boosting model) to examine the relationships between nonlinear features and SRH associations; we also used partial dependence plots to explore the potential threshold effect of factors on older adults’ SRH. The results indicate that older adults lacking medical insurance might be burdened by out-of-pocket expenses stemming from the high cost of medical expenses. BMI and personal medical expenditure play important roles in predicting older adults’ SRH, with a BMI threshold range of 20–27 potentially predicting good SRH; similarly, a personal annual medical expenditure threshold of 11,000 yuan predicts poor SRH. Policy-makers might need to further adjust healthcare and health insurance systems so as to alleviate the financial burdens that come with illness and improve overall accessibility to healthcare services.

## Figures and Tables

**Table 1:** Baseline factors examined in this study. SD: standard deviation.

Variable	Definition	Mean	SD
Self-rated health	1 = Good SRH, 0 = Poor SRH	0.721	0.448
Age (Age > =60)	Continuous variables (years)	66.657	5.241
Gender	1 = Male, 0 = Female	0.571	0.495
Urban	1 = Urban residents, 0 = Rural residents	0.365	0.481
Education	1 = Junior high school and above, 0 = otherwise	0.701	0.458
Income	Total income, continuous variables (yuan)	3624.729	13,664.587
Medical expenditure	Total personal expenditure on medical, continuous variables (yuan)	4676.916	15,681.112
Marital Status	1 = Married, 0 = otherwise	0.870	0.336
Hukou	1 = Urban hukou, 0 = non-urban hukou	0.166	0.372
Employment	1 = employed, 0 = otherwise	0.777	0.416
Smoke	1 = Current smoker, 0 = non-current smoker	0.499	0.500
Drink	1 = Current drinker, 0 = non-current drinker	0.207	0.405
Exercise	Frequency of physical exercise, continuous variables (times)	3.167	3.600
Retirement	1 = retiree, 0 = otherwise	0.113	0.316
Insurance	1 = respondents had medical insurance, 0 = otherwise	0.652	0.476
Satisfaction with level of medical	The level of medical expertise, Ordinal variable 1 (very bad) to 5 (very good)	3.610	0.914
Chronic	1 = Had a chronic disease, 0 = no chronic disease	0.281	0.450
Family size	Continuous variables	3.987	2.168
Attitude toward medical service problem	Ordinal variable, (0) no problem to (10) extremely serious problem	5.991	2.958
Attitude toward security problem	Ordinal variable, (0) no problem to (10) extremely serious problem	5.400	3.010
Trust in doctors	Ordinal variable, (0) distrustful to (10) very trusting	7.022	2.471
BMI	Continuous variables	22.832	3.605

**Table 2:** Importance of independent variables in predicting older adults’ SRH by F-score and SHAP.

Predictors	Ranking of Feature Importanceby F-Score	Ranking of Feature Importance by SHAP
Related health predictors		
BMI	1	3
Chronic disease	16	2
Smoke	13	20
Drink	19	13
Physical exercise	9	7
Medical expenditure and perceived medical attitudes		
Personal medical expenditure	2	1
Attitude toward medical service problem	4	11
Attitude toward social security problem	5	9
Trust in doctors	6	12
Satisfaction with level of medical expertise	8	16
Sociodemographic predictors		
Age	3	6
Gender	14	4
Marital status	18	21
Educational attainment level	12	19
Household total income level	10	5
Family sizes	7	17
Employment status	17	8
Hukou status	20	10
Retirement status	21	15
Insurance	11	18
Urban	15	14

Notes: SHAP feature importance was calculated using the SHAP explainer that ran based on the trained XGBoost model.

## Data Availability

Original data can be accessed through https://opendata.pku.edu.cn/dataverse/CFPS?q=&types=files&sort=dateSort&order=asc (27 October 2021)**.** upon receiving permission from Peking University Open Research Data.
